# Exposure to Zoonotic West Nile Virus in Long-Tailed Macaques and Bats in Peninsular Malaysia

**DOI:** 10.3390/ani10122367

**Published:** 2020-12-10

**Authors:** Mohd Yuseri Ain-Najwa, Abd Rahaman Yasmin, Siti Suri Arshad, Abdul Rahman Omar, Jalila Abu, Kiven Kumar, Hussni Omar Mohammed, Jafar Ali Natasha, Mohammed Nma Mohammed, Faruku Bande, Mohd-Lutfi Abdullah, Jeffrine J. Rovie-Ryan

**Affiliations:** 1 Department of Veterinary Laboratory Diagnosis, Faculty of Veterinary Medicine, Universiti Putra Malaysia, UPM Serdang, Selangor 43400, Malaysia; ainnajwa2111@gmail.com (M.Y.A.-N.); natashali036@gmail.com (J.A.N.); mohdnma@live.com (M.N.M.); 2 Laboratory of Vaccines and Biomolecules, Institute of Bioscience, UPM Serdang, Selangor 43400, Malaysia; aro@upm.edu.my; 3 Department of Veterinary Pathology and Microbiology, Faculty of Veterinary Medicine, Universiti Putra Malaysia, UPM Serdang, Selangor 43400, Malaysia; suri@upm.edu.my; 4 Department of Veterinary Clinical Studies, Faculty of Veterinary Medicine, Universiti Putra Malaysia, UPM Serdang, Selangor 43400, Malaysia; jalila@upm.edu.my; 5 Department of Pathology, Faculty of Medicine and Health Sciences, Universiti Putra Malaysia, UPM Serdang, Selangor 43400, Malaysia; kivenkumar@yahoo.com; 6 Department of Population Medicine and Diagnostic Sciences, Cornell University, Ithaca, NY 14853, USA; hussni.mohammed@cornell.edu; 7 Department of Veterinary Services, Ministry of Animal Health and Fisheries Development, Sokoto 840, Sokoto State, Nigeria; bandeyabo@gmail.com; 8 Department of Conservation of Biodiversity of Wildlife and National Park Malaysia, Ministry of Energy and Natural Resources, Kuala Lumpur 56000, Malaysia; lutfi@wildlife.gov.my (M.-L.A.); jeffrine@wildlife.gov.my (J.J.R.-R.)

**Keywords:** west nile virus, arbovirus, zoonotic, macaque, bats, c-ELISA, RT-PCR

## Abstract

**Simple Summary:**

The role of wildlife animals, such as macaques and bats, in the spreading and maintenance of deadly zoonotic pathogens in nature are documented in several studies. The present study substantially highlights the first evidence of West Nile Virus (WNV) infection, a mosquito borne virus in the Malaysian macaques and bats. Of the 81 macaques sampled, 24 of the long-tailed macaques were seropositive to WNV, indicating that they were exposed to the virus in the past. The long-tailed macaques were found in the mangrove forests located in the Central, Southern, and West Peninsular Malaysia. Meanwhile, five out of 41 bats (Lesser Short-nosed Fruit Bats, Lesser Sheath-tailed Bats, and Thai Horseshoe Bats) that were found in the caves from Northern Peninsular Malaysia showed susceptibility to WNV. Therefore, a constant bio surveillance of WNV in the wildlife in Malaysia is a proactive attempt. This study was aligned with the Malaysian government’s mission under the Malaysia Strategy for Emerging Diseases and Public Health Emergencies (MYSED) II (2017–2021) and the Ministry of Health priorities in order to enhance the regional capability to rapidly and accurately survey, detect, diagnose, and report outbreaks of pathogens and diseases of security concern.

**Abstract:**

The role of wildlife such as wild birds, macaques, and bats in the spreading and maintenance of deadly zoonotic pathogens in nature have been well documented in many parts of the world. One such pathogen is the mosquitoes borne virus, namely the West Nile Virus (WNV). Previous research has shown that 1:7 and 1:6 Malaysian wild birds are WNV antibody and RNA positive, respectively, and bats in North America may not be susceptible to the WNV infection. This study was conducted to determine the status of WNV in Malaysian macaques and bats found in mangrove forests and caves, respectively. Archive sera and oropharyngeal swabs from long-tailed macaques were subjected to the antibody detection using WNV competitive enzyme-linked immunosorbent assay (c-ELISA) and WNV RNA using RT-PCR, respectively, while the archive oropharyngeal and rectal swabs from bats were subjected to RT-PCR without serological analysis due to the unavailability of serum samples. The analysis revealed a WNV seropositivity of 29.63% (24/81) and none of the macaques were positive for WNV RNA. Meanwhile, 12.2% (5/41) of the bats from Pteropodidae, Emballonuridae, and Rhinolophidae families tested positive for WNV RNA. Here, we show a high WNV antibody prevalence in macaques and a moderate WNV RNA in various Malaysian bat species, suggesting that WNV circulates through Malaysian wild animals and Malaysian bat species may be susceptible to the WNV infection.

## 1. Introduction

Deadly emerging and re-emerging zoonotic pathogens are transmitted mostly from wildlife reservoirs to humans or other animals during spillover events, with or without a vector intervention. Evidence has shown that some of the medically important mosquito borne illnesses causing West Nile fever, dengue, malaria, chikungunya, zika, and Japanese encephalitis were isolated from the wildlife [1,2,3]. The West Nile fever is distributed in Africa, USA, Europe, and Western Asia causing febrile illness and encephalitis in humans and animals [4]. The causative agent is an envelope RNA virus known as the West Nile Virus (WNV), which is classified in the genus of *Flavivirus* under the family of Flaviviridae [5]. Despite the role played by wild birds as amplifier hosts of WNV and mosquitoes as vectors, the role of wildlife such as macaques and bats in the WNV transmission cycle remains poorly understood [6].

Several epidemiological studies attested that the macaque species could become infected with WNV [6,7,8]. Nevertheless, the macaques developed a low level of viraemia based on the experimental study, making them unlikely to perpetuate the virus [8]. Meanwhile, bats have been demonstrated as a competent amplifying host of arthropod borne Flaviviruses transmission, but have not been proven to maintain WNV infections as seen in the North American bats, which were infrequently infected with WNV [9,10]. Due to the nature of wildlife habitats and constant exposure to mosquito bites during blood meals, there is an abundant considerable opportunity for WNV introduction to the wildlife. There is also the possibility of spillover events as well as the sylvatic transmission of the virus from the wildlife to humans due to human activities such as deforestation and urbanization, and also due to other climate-related factors that lead to a loss of wildlife habitat [11,12].

In Malaysia, some studies have reported evidence of exposure of humans, birds, and mosquitos to WNV. A study by Marlina et al. [13] reported a WNV seroprevalence of 1.21% (9/742) in the Orang Asli from several states in Peninsular Malaysia. Additionally, Rais et al. [14] and Ain-Najwa et al. [15] reported a WNV seroprevalence of 4.41% (3/68) in captive birds and 18.71% (29/155) in wild birds, respectively. In 1970, a sub-type of WNV designated as the Kunjin Virus (KUNV), which was originally endemic in Australia was detected in *Culex pseudovishnui* mosquitoes in Sarawak, a Malaysian state of Borneo [16]. More recently, WNV was also detected in pooled samples of *Culex* spp. mosquitoes, which were trapped close to the migratory birds landing areas in Malaysia (under review). These findings collectively demonstrated evidence of WNV exposure with asymptomatic infection in animals and humans in Malaysia [17,18]. Nevertheless, the status of WNV infection in Malaysian macaques and bats remains poorly understood. Therefore, this study was conducted to determine the serological and/or molecular prevalence of WNV in bats and macaques in selected areas of Malaysia.

## 2. Materials and Methods

### 2.1. Ethical Statement

All experimental procedures involving the archived samples that originated from the Department of Wildlife and National Parks (DWNP) were conducted in accordance with guidelines approved by DWNP, Malaysia with the research permit number JPHL&TN (IP):100-6/1/14. The no. IACUC approval was needed in this study, since the archive samples were used

### 2.2. Study Design

The archived macaque and bat samples originating from several states in Peninsular Malaysia were shared by the Department of Conservation of Biodiversity of Wildlife and National Park Malaysia. A total of 88 long-tailed macaques and 41 bats, which were sampled from the year 2014 to 2017, were included in this study. The samples obtained from macaques included 81 sera and 63 oropharyngeal swabs, while 38 rectal swabs and 34 oropharyngeal swabs were obtained from bats. Sera and swabs samples (oropharyngeal and rectal) were subjected to serological and molecular analysis, respectively. Since the archive samples were used, the samples obtained in this study were based on availability. Consequently, an inconsistent number of samples relative to the total number of animals were observed in this study. In addition, due to the unavailability of bats sera samples, no serological analysis was performed for bats.

**Figure 1:**
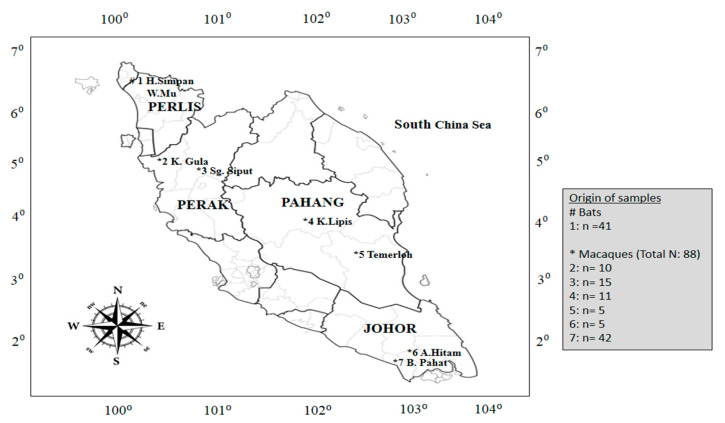
Map of Peninsular Malaysia showing the origin of bat and macaque samples. Note: The hash (#) symbol indicates the origin of bat samples, which were collected from (1) Hutan Simpan Wang Mu located in Perlis state (Northern Malaysia), while the asterisk (*) symbol indicates the origin of macaque samples from (2) Kuala Gula and (3) Sungai Siput located in Perak state (West Coast Malaysia); (4) Kuala Lipis and (5) Temerloh Pahang state (central Malaysia); and (6) Ayer Hitam and (7) Batu Pahat from Johor state (Southern Malaysia). N: Total animals; n: No. of animals.

Macaques were sampled from mangrove forests in Pahang state (central Peninsular Malaysia), Perak state (West Coast Peninsular Malaysia), and Johor state (Southern Peninsular Malaysia) (Figure 1). In Pahang state, the macaques were captured at (1) Kuala Lipis (4.1843° N, 102.0542° E) and (2) Temerloh (3.4486° N, 102.4163° E); in Perak state, they were captured at (3) Sungai Siput (4.8190° N, 101.0737° E) and (4) Kuala Gula (4.933° N, 100.467° E), while in Johor state, they were captured at (5) Ayer Hitam (1.9183° N, 103.1800° E) and (6) Batu Pahat (1.8469° N, 102.9352° E). Meanwhile, bats were sampled from a cave located in (7) Hutan Simpan Wang Mu in Perlis State Park (6.467° N, 100.250° E) from Perlis state (Northern Peninsular Malaysia) (Figure 1). Both sites are a natural habitat for macaques and bats found in Malaysia.

### 2.3. Serological Analysis

The status of WNV seropositivity in macaques was determined using a commercial WNV IgG antibody-based c-ELISA kit (ID Screen West Nile Competition Multi-species ELISA, ID VET, Montpellier, France) pre-coated with the WNV envelope protein (prE) from macaque’s sera. However, the kit cross-reacted with other Flavivirus namely the Japanese Encephalitis Virus (JEV) and Yellow Fever. Since Yellow Fever is not endemic in Malaysia, sera were subjected to JEV screening using a specific double-antibody sandwich ELISA (DAS-ELISA) (Sun red, Shanghai, China) to rule out cross-reactivity.

For the WNV ELISA analysis, the positive and negative controls were run in duplicate experiments. If the sample showed a percentage of S/N (S: Sample optical density (OD); N: Negative control OD) less than or equal to 40%, the reaction was considered positive. In this study, the prevalence of WNV antibodies in macaques was calculated as a percentage of positive samples over the total number of examined samples at a 95% confidence interval. Data obtained in this study could not be analyzed for risk factors due to the uneven number of samples from different locations, which may have led to a potential bias.

### 2.4. Reverse Transcriptase—Polymerase Chain Reaction (RT-PCR) Assay

Oropharyngeal and rectal swabs were subjected to total RNA extraction using TRIsure (Bioline, London, UK) according to the manufacturer’s instructions. The concentration and purity of the RNA extracted were determined using a BioPhotometer (Eppendorf, Hamburg, Germany). Synthetic plasmid was used as a positive control and for the RT-PCR primer set which targeted highly conserved regions between WNV Capsid (C) and Pre-Membrane (prM) proteins, as previously described by Ain-Najwa et al. [15] (Table S1). The one-step RT-PCR using MyTaq (Bioline, Memphis, TN, USA) in a total of 25 μL reaction was performed, as previously described by Ain-Najwa et al. [15]. Gel electrophoresis was conducted to view the amplification of positive reactions visualized by the presence of a 470-bp amplicon fragment between C and prM genes aligned with a positive control band.

### 2.5. DNA Sequencing and Bioinformatic Analysis

The DNA sequencing analysis was performed by purifying the band using a gel purification kit (Nucleospin Gel and PCR Clean-up kit (Macherey-Nagel, Duren, Germany) and sequenced in both directions using gene specific primers in the ABI PRISM 3730xl Genetic Analyzer (Applied Biosystems, Foster City, CA, USA). The sequences obtained were searched using the Basic Local Alignment Search Tool (BLAST) algorithm (https://blast.ncbi.nlm.nih.gov/Blast.cgi). A total of 63 sequences of WNV (Table S2) including 16 sequences based on a previous study [15] were included in the sequences alignment using Multiple Sequence Alignment (MAFFT) software version 7. The phylogenetic tree was constructed using neighbor-joining with the Maximum Composite Likelihood model in Molecular Evolutionary Genetics Analysis (MEGA) 7 [19]. A bootstrapped confidence interval with 1000 replicates was set. The Newick file was created and the tree was viewed and edited using the Interactive Tree Of Life (iTOL). The percentage identity of each nucleotide and amino acids sequences of WNV sequences used in the phylogenetic tree were subjected into a pairwise distance by MEGA-7.

## 3. Results

### 3.1. WNV Antibodies in Macaques

The serological analysis of WNV using c-ELISA revealed that 24 out of 81 macaques (29.63% (24/81) at 95% CI (0.203 to 0.410)) were seropositive with a S/N% (S: Sample OD; N: Negative control OD) value less than or equal to 40%. All of these serum samples were further analyzed using JEV DAS-ELISA and none of the samples showed a positive reaction towards JEV. The distribution of the WNV positive antibody in macaques according to the sample origin, age, and sex is provided in Table 1.

Among the three states sampled, 14 macaques from Johor showed the highest positive WNV followed by eight macaques from Perak and the remaining two from Pahang. Of the total WNV seropositive macaques, 16 were male and eight were female. Furthermore, adult macaques showed the highest WNV seropositive number with 17 followed by seven macaques from the juvenile group.

### 3.2. Molecular Analysis of West Nile Virus in Bats

The one-step RT-PCR revealed that 12.2% (5/41) of the bats tested positive for WNV RNA, while none of the macaques were positive. The distribution of positive WNV RNA in bats from the present study is shown in detail in Table 2. Of the five bat families studied, WNV positive bats were detected from Pteropodidae, Emballonuridae, and Rhinolophidae families. With regard to species, the highest number of positive bats was from *Emballonura monticola* (three bats positive) followed by *Cynopterus brachyotis* (one bat positive), and *Rhinolophus siamensis* (one bat positive). Both oropharyngeal and rectal swabs from one *R. siamensis* bat showed a positive WNV RNA in RT-PCR analysis, while the other bats were positive either from oropharyngeal or rectal swabs. Of the five positive bats, three were male, while two were female bats. Among them, three out of five bats were adult, while the remaining two were juvenile.

**Figure 2:**
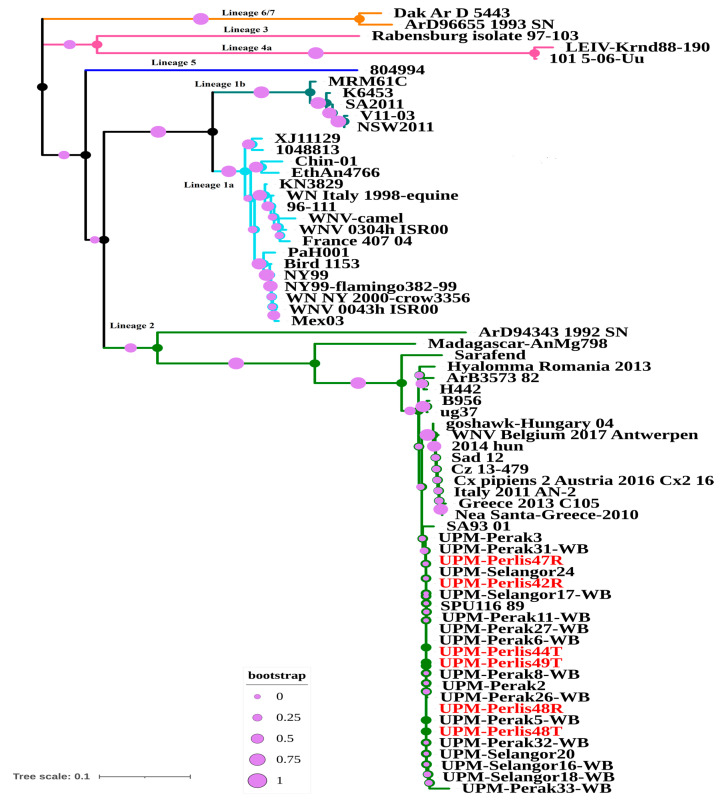
Phylogenetic analysis of partial sequence between capsid (C) and pre-membrane (prM) genes of West Nile Virus detected in bats in Malaysia with other global WNV isolates. The analysis involved 69 nucleotide sequences including six sequences from this study (highlighted in red), which have a 470-bp nucleotide length obtained from the bats. The tree branches were colored by the proposed WNV lineages; orange: Lineage 6/7; hibiscus: Lineage 3 and lineage 4a; blue: Lineage 5; dark cyan: Lineage 1b; sky blue: Lineage 1a; and green: Lineage 2. All the WNV sequences detected from bats are identical to the previous study, which was detected in migratory birds and the SPU 116 98 strain from South Africa. Branches are scaled bootstrap.

Six positive sequences were submitted to the GenBank under the following accession numbers: MK327803–MK327808. The sequencing analysis revealed that isolates from the present study showed a 99.25–100% similarity with the SPU116/89 strain. The pairwise percentage identity between the nucleotide and amino acid of selective WNV strains sequences with local isolates from bats showed a range of high, medial, and low similarity. The highest identical percentage was shown by the SPU116_89 strain (a human strain), while the Rabensburg isolate 97–103, Dak Ar D 5443, ArD96655/1993/SN, LEIV-Krnd88-190, and 101_5-06-Uu strains were less similar (Figure S1). A phylogenetic tree constructed using neighbor-joining methods, showing evolutionary relationships of taxa of WNV positive isolates from this study, were grouped together with WNV strains from the WNV lineage 2 (Figure 2).

## 4. Discussion

During the mosquito breeding seasons, there is an increase in the reported cases of West Nile Virus outbreaks in the USA, India, and Europe, involving humans and horses [20]. However, in tropical countries such as Malaysia, that have hot, humid, and monsoon seasons, the active mosquito breeding occurs throughout the year [21]. The infection rate is substantial since mosquitoes readily transmit deadly viruses during blood feeding. Although there is no evidence of WNV outbreaks in Malaysia thus far, unprecedented outbreaks of the WNV neuro-invasive disease leading to deaths in humans and animals have occurred elsewhere, due to the lack of early detection and intervention

Apart from mosquito vector control programmes, the WNV surveillance in wildlife is essential for elucidating the status of WNV shedding, with the aim of preventing a virus spillover into the humans and animals population. In the WNV infection, wild birds are a prominent WNV amplifier and reservoir [22]. Nevertheless, the role of wildlife animals, such as Malaysian macaques and bats are unknown. By considering this fact, the bio surveillance of the WNV antibody and RNA were conducted in archived sera and swab samples. The exposure to WNV was determined using a WNV IgG based competitive ELISA. The gold standard to confirm the presence of the WNV antibody was achieved by the neutralization test [23], however, it was not performed in this study due to the unavailability of the high containment biosafety facility required for the test. To rule out the cross reactivity with other Flavivirus species, a specific JEV based ELISA was included in the present study.

This study indicated that 29.63% (24/81) of long-tailed macaques were seropositive towards WNV. Although WNV RNA were not detected in macaques, they have been previously exposed to the virus as evidenced by the presence of the WNV IgG-antibody. This seropositive rate could possibly be due to the natural proximity between *Culex* mosquitoes and the macaque species within the forest habitat, thus causing WNV-specific antibodies to remain longer in macaques [8,24]. Several studies from around the world have reported a varied WNV seroprevalence rate ranging from low to moderate in macaques. In Louisiana, the WNV seroprevalence of 51.4%, 39.4%, 20.3%, 12.5% (2/16), and 6.6% (3/45) were detected in captive baboons (*Papio* spp.), rhesus macaques (*Macaca mulatta*), southern pig-tailed macaques (*Macaca nemestrina*), Japanese macaques (*Macaca fuscata*), and Georgian sooty mangabeys (*Cercocebusatys*), respectively [7,25,26].

Defining exactly the vulnerability to the WNV infection is among the most crucial indicators to be studied for effective WNV control and preventive measures. Therefore, oropharyngeal and rectal swabs obtained from bats were subjected to the one-step RT-PCR and partial sequencing targeting the WNV genes between the capsid and pre-membrane. In the present study, bats from the Pteropodidae, Emballonuridae, and Rhinolophidae families were shown to shed WNV. Among these three families, Pteropodidae and Rhinolophidae were found to be the reservoir for the Nipah Virus (*Paramyxovirus*) and SARS-CoV (Coronaviridae) [27,28,29]. As evidenced by this study, bats might possibly be infected with WNV either from blood-feeding mosquitoes or directly from mosquito ingestion, as bats are insectivores [30].

Thus far, lineage 1 and 2 have been associated with profound effects during outbreaks in humans [31]. The partial sequencing analysis from the present study demonstrated that the bats shed WNV from lineage 2, particularly from SPU116/89 strains from South Africa. This strain was previously recovered from a liver of human fatal hepatitis in South Africa. The same strain has been discovered in equine in Africa and showed an extreme neuro-invasive effect in experimental mice [32,33]. Interestingly, WNV lineage 2 from the same strain was also found in migratory and water birds found in Malaysia based on a recent study [18]. Further sequencing of the whole virus genome, or at least the hypervariable domain may possibly explain the intercontinental relatedness of WNV isolates from Malaysia and that of South Africa. Ecological links such as migratory factors could also provide a possible translocation of the virus or provide a link between bats in South Africa and Asian regions.

The current study demonstrates a relatively moderate prevalence rate of WNV in macaques and bats despite having a smaller sample size even in the first attempt of the study. This might be due to the natural exposure to Flavivirus in the wildlife mammals [34]. The pronounced exposure of the Malaysian wildlife to the mosquito-borne illness is anticipated, since a single species of mosquitoes exhibits the ability to carry more than one pathogen. For example, *Culex* spp. has been well recognized as the main vector for WNV transmission, as well as Chikungunya and JEV [35,36,37]. Moreover, staying in an ecosystem that favors mosquito breeding such as a mangrove forest or cave engenders them constantly to be exposed to mosquito bites [38]. Although the exact weather of the actual sampling period is unknown, it is believed that tropical countries such as Malaysia with a rainy, hot, and humid climate contribute to the high population of mosquitoes from the origin of the samples obtained. Collectively, contributing factors such as climate, ecosystem, habitat loss, antigenic properties of the virus, and the availability of vectors and host favor Malaysia to be prevalent to mosquito-borne illness.

## 5. Conclusions

To summarize, this study substantially highlights the first evidence of WNV infection in bats and long-tailed macaques in Malaysia. Therefore, a constant bio surveillance screening of WNV in the wildlife in Malaysia is a proactive attempt. This study was aligned with the Malaysian government’s mission under the Malaysia Strategy for Emerging Diseases and Public Health Emergencies (MYSED) II (2017–2021) under the Ministry of Health priorities in order to enhance the regional capability to rapidly and accurately survey, detect, diagnose, and report outbreaks of pathogens and diseases of security concern.

## Figures and Tables

**Table 1:** West Nile Virus (WNV) c-ELISA and RT-PCR results in long-tailed macaques according to states, sex, age, and type of sample.

Family	Species	Malaysian State	No. of Macaques	No. of Serum Sample	No. of Positive WNV Antibody (c-ELISA)	No. of Oropharyngeal Swabs	No. of Positive WNV RNA (RT-PCR)
*Cercopithecidae*	Long-tailed macaque (*Macaca Fascicularis)*	Pahang	8	6	1	8	0
			0	0	-	0	-
			4	2	1	4	0
			0	0	-	0	-
			0	0	-	0	-
			4	1	0	3	0
		Perak	5	5	3	0	-
			0	0	-	0	-
			11	11	2	1	0
			5	5	2	0	-
			0	0	-	0	-
			4	4	1	0	0
		Johor	5	5	1	5	0
			3	3	0	3	0
			21	21	8	21	0
			6	6	0	6	0
			2	2	0	2	0
			10	10	5	10	0
Total			88	81	24	63	0

M: Male; F: Female; c-ELISA: Competitive ELISA; RT-PCR: Reverse transcriptase-PCR. Adult ≥ 5 years; sub adult ≥ 3–5 years, and juvenile ≥ 1–3 years old.

**Table 2:** West Nile Virus RNA detection in bats according to families, species, sex, age, and type of sample.

Family	Species	No. of Bats	No. of Oropharyngeal Swabs	No. of Rectal Swabs	No. of Positive WNV RNA (RT-PCR)
Pteropodidae	Horsfield’s Fruit Bats (*Cynopterus horsfieldii)*	1	1	1	0
		5	4	5	0
		2	2	2	0
		1	1	1	0
	Lesser Short-nosed Fruit Bats (*Cynopterus brachyotis*)	0	0	0	-
		0	0	0	-
		1	1	1	1
		0	0	0	-
	Long-tongued Fruit Bats (*Macroglossus sobrinus*)	0	0	0	-
		1	1	1	0
		0	0	0	-
		0	0	0	-
Emballonuridae	Lesser Sheath-tailed Bats (*Emballonura monticola*)	0	0	0	-
		3	3	3	2
		1	1	1	0
		2	2	2	1
Rhinolophidae	Blyth’s Horseshoe Bats (*Rhinolophus lepidus*)	1	1	1	0
		2	1	2	0
		5	1	5	0
		2	1	2	0
	Malayan Horseshoe Bats (*Rhinolophus malayanus*)	0	0	0	0
		2	2	2	0
		0	0	0	0
		0	0	0	0
	Thai Horseshoe Bats (*Rhinolophus siamensis*)	1	1	1	2
		0	0	0	-
		0	0	0	-
		0	0	0	-
	Croslet Horseshoe Bats (*Rhinolophus coelophyllus*)	0	0	0	-
		1	1	1	0
		0	0	0	-
		0	0	0	-
	Bourret’s Horseshoe Bats (*Rhinolophus paradoxolophus)*	0	0	0	-
		0	0	0	-
		0	0	0	-
		1	1	1	0
Hipposideridae	Great Roundleaf Bats (*Hipposideros armiger*)	1	1	1	0
		0	0	0	-
		0	0	0	-
		1	1	1	0
	Shield-faced Roundleaf Bats (*Hipposideros lylei*)	1	1	0	0
		1	1	1	0
		2	2	1	0
		1	1	0	0
	Diadem Leaf-nosed Bats (*Hipposideros diadema*)	0	0	0	-
		1	1	1	0
		0	0	0	-
		0	0	0	-
Vespertilionidae	Lesser Asiatic Yellow Bats (*Scotophilus kuhlii*)	0	0	0	-
		1	1	1	0
		0	0	0	-
		0	0	0	-
TOTAL		41	34	38	Bats +ve: 5 RT-PCR +ve: 6

M: Male; F: Female; c-ELISA: Competitive ELISA; RT-PCR: Reverse transcriptase-PCR; O: Orophryngeal swab; R: Rectal. Juvenile < 9 months; Adult > 9 months.

## Supplementary Materials

The following are available online at https://www.mdpi.com/2076-2615/10/12/2367/s1, Table S1: Sequence and location used for RT-PCR to amplify WNV gene, Table S2: List of references strains used in the phylogenetic and pairwise analysis. Figure S1: Pairwise percent identity between nucleotide (black) and amino acid (blue) sequences of selective WNV strains with local detection. The identity percentage range was set from high, medial and low similarity as indicated by light green, yellow and red color, respectively.
