# Supplementary material for: Exposure to Zoonotic West Nile Virus in Long-Tailed Macaques and Bats in Peninsular Malaysia
Source: Animals (Basel). 2020 Dec 10;10(12):2367. doi: 10.3390/ani10122367 (PMC7764493; doi:10.3390/ani10122367)
Supplement: Supplementary file 1 [file animals-10-02367-s001.pdf]

Supplementary Table S1: Sequence and location used for RT-PCR to amplify WNV gene.

Primers	Sequence	Nucleotide Location 5' to 3'	Product Size (bp)
Forward	5' CCA ATA CGT TTC GTG TTG G 3'	222-241	470
Reverse	5' ATG TCT TCA GGG TCA TTT CC 3'	659-639	

Supplementary Table S2: List of references strains used in the phylogenetic and pairwise analysis.

No.	Strains name	GenBank accession no	No	Strains name	GenBank accession no
1	SA93/01	EF429198.1	36	Cx. pipiens 2/Austria/2016 (Cx2/16	MF984352.1
2	goshawk-Hungary/04	DQ116961.1	37	Sarafend	AY688948.1
3	Italy/2011/AN-2	jn858070.1	38	Madagascar-AnMg798	DQ176636.2
4	Nea Santa-Greece-2010	hq537483.1	39	Rabensburg isolate 97-103	AY765264.1
5	Sad/12	kc407673.1	40	804994	DQ256376.1
6	ug37	NC_001563.2	41	LEIV-Krnd88-190	AY277251.1
7	K6453	GQ851603.1	42	Dak Ar D 5443	EU082200.2
8	NSW2011	JN887352.1	43	SA2011	KT934803.1
9	ArD96655/1993/SN	KY703855.1	44	V11-03	JX123030.1
10	ArD94343/1992/SN	KY703856.1	45	MRM61C	KX394398.1
11	UPM-Selangor20	MK327790.1	46	Chin-01	AY490240.2
12	UPM-Selangor24	MK327791.1	47	EthAn4766	AY603654.1
13	UPM-Perak2	MK327792.1	48	WN Italy 1998-equine	AF404757.1
14	UPM-Perak3	MK327793.1	49	France 407/04	DQ786572.1
15	UPM-Perak5	MK327794.1	50	96-111	AY701412.1
16	UPM-Perak6	MK327795.1	51	KN3829	AY262283.1
17	UPM-Perak8	MK327796.1	52	WNV_0304h_ISR00	HM152775.1
18	UPM-Perak11	MK327797.1	53	WNV-camel	KU588135.1
19	UPM-Perak26	MK327798.1	54	NY99-flamingo382-99	AF196835.2
20	UPM-Perak27	MK327799.1	55	NY99	NC_009942.1
21	UPM-Perak31	MK327800.1	56	WN NY 2000-crow3356	AF404756.1
22	UPM-Perak32	MK327801.1	57	Bird 1153	AY712945.1
23	UPM-Perak33	MK327802.1	58	Mex03	AY660002.1
24	UPM-Selangor17	MK327788.1	59	WNV_0043h_ISR00	HM152773.1
25	UPM-Selangor16	MK327787.1	60	PaH001	AY268133.1
26	UPM-Selangor18	MK327789.1	61	XJ11129	JX442279.1
27	SPU116/89	EF429197.1	62	1048813	KC601756.1

28	Hyalomma/Romania/2013	KJ934710.1	63	101_5-06-Uu	FJ159129.1
29	ArB3573/82	DQ318020.1	64	Perlis49T	MK327808
30	H442	EF429200.1	65	Perlis48T	MK327807
31	2014/hun	KT359349.1	66	Perlis48R	MK327806
32	B956	AY532665.1	67	UPM-Perlis47R	MK327805
33	WNV/Belgium/2017/Antwerpen	MH021189.1	68	UPM-Perlis44T	MK327804
34	Cz 13-479	KM203862.1	69	UPM-Perlis42R	MK327803
35	Greece/2013/C105	KJ577738.1			

Positive West Nile virus detected from this study is highlighted in red.

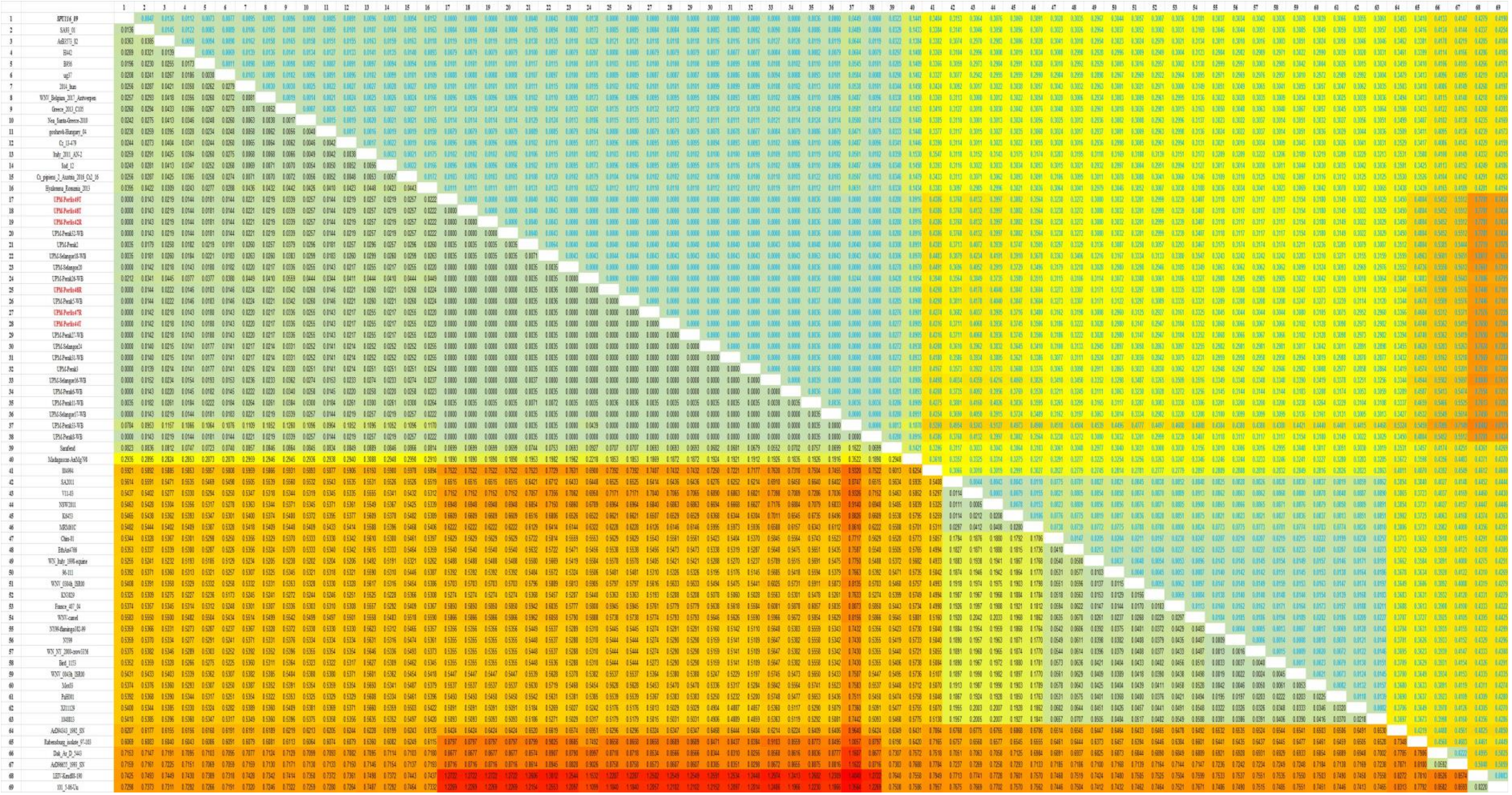

Supplementary Figure S1: : Pairwise percent identity between nucleotide (black) and amino acid (blue) sequences of selective WNV strains with local detection. The identity percentage range was set from high, medial and low similarity as indicated by light green, yellow and red color, respectively.
